# Identifying the knowledge needs and preferences of parents of children with rare diseases regarding clinical trials: a scoping review protocol

**DOI:** 10.1186/s13643-026-03094-0

**Published:** 2026-02-05

**Authors:** Annie P. Mabbott, Lisa Knisley, Shannon D. Scott

**Affiliations:** 1 Faculty of Nursing, University of Alberta, Level 3, Edmonton Clinic Health Academy, 11405 87 Avenue, Edmonton, AB T6G 1C9 Canada; 2 College of Nursing, Rady Faculty of Health Sciences, Helen Glass Center for Nursing, University of Manitoba, 89 Curry Place, Winnipeg, MB R3T 2N2 Canada; 3 Stollery Children’s Hospital Foundation, Level 3, Edmonton Clinic Health Academy, 11405 87 Avenue, Edmonton, AB T6G 1C9 Canada

**Keywords:** Rare disease, Pediatrics, Parents, Clinical trials, Knowledge needs, Information needs, Knowledge translation, Scoping review

## Abstract

**Background:**

Rare diseases (i.e., incidence of <1/2000) are individually uncommon, but collectively these 10,000 conditions affect an estimated 473 million people globally, and approximately 70% of rare diseases manifest in childhood. Despite this global impact, 90% of rare diseases lack effective treatment. Treatments for rare diseases are often identified through clinical trials. Identifying parents’ knowledge needs and preferences regarding pediatric rare disease clinical trials is an important aspect of empowering parents, improving clinical research practices, and potentially improving recruitment to these vital trials. The aim of the scoping review is to determine the extent, range, and characteristics of the evidence on the knowledge needs and preferences of parents regarding pediatric rare disease clinical trials.

**Methods:**

A scoping review will be conducted to identify sources of literature on the topic. A systematic search strategy co-developed with a research librarian will be conducted in six databases (Medline, EMBASE, CINAHL, Scopus, Web of Science, and PsycINFO). Gray literature will be searched via Google, Perplexity AI, the ProQuest Dissertations & Theses Global database, and relevant rare disease organizational websites. Abstract and full-text screening will be conducted by two reviewers independently. Studies in English will be included regardless of study design, date of publication, or location of study/publication. Study quality will be appraised using the Mixed Methods Appraisal Tool. Data will be extracted including study characteristics, population, phenomena under investigation, and knowledge needs and preferences identified. Analysis will involve a descriptive numerical summary and qualitative content analysis. Findings will be presented in evidence tables, and patterns, themes, and gaps across the data will be reported using a narrative approach.

**Discussion:**

This review will provide an overview of the existing literature regarding parents’ knowledge needs and preferences about pediatric rare disease clinical trials. The findings of this review will inform future research and the development of knowledge translation resources for parents of children with rare diseases.

**Systematic review registration:**

This protocol has been registered in Open Science Framework (registration: https://doi.org/10.17605/OSF.IO/QXR8G).

**Supplementary Information:**

The online version contains supplementary material available at 10.1186/s13643-026-03094-0.

## Background

Rare diseases (RDs) are diseases that affect a small number of people compared to the general population (i.e., prevalence of <1/2000) [1]. Although individually rare, collectively there are approximately 10,000 RDs, affecting an estimated 473 million people globally [2, 3]. RDs are complex and are often progressive, chronic conditions that can result in debilitating impairments, and many are life-threatening [4].

Approximately 70% of RDs manifest in childhood [5]. Children with these conditions account for a quarter of pediatric inpatient beds in Canada, requiring complex medical care from diagnosis through treatment [4]. Despite the significant number of RDs and associated global impact, it is estimated that over 90% of RDs still lack effective treatment [6].

In Canada, individuals with RDs frequently experience delays and difficulties in accessing treatments [7], which include targeted drugs and technologies, as well as gene-based, cell-based, and small molecule therapies [6]. A current challenge for accessing treatments for children with RDs in Canada is unnecessary, inefficient, and costly barriers to initiating and conducting CTs; this includes a lack of natural history data, inadequate sample sizes (due to low prevalence), and prohibitive costs [4]. Due to various economic, regulatory, and policy factors, only 60% of treatments for RDs are available in Canada, and most get approved up to 6 years later than in Europe and the USA [7, 8]. There is an urgent need to simplify and speed up access to treatments for children with RDs. RareKids-CAN [9] is a national network dedicated to developing and executing high-quality pediatric RD CTs (PRDCTs) and improving access to therapies for children in Canada. CTs are essential for advancing medical knowledge and identifying treatment options for RDs.

The diagnostic journey for RDs can be lengthy and agonizing for families, and treatments (when available) are often very expensive [4]. The burden and challenges associated with RDs often fall on the child’s family members, particularly their parents. Family members of a child with a RD can experience significant psychosocial impacts (including psychological distress, lower quality of life, and higher caregiver burden), negative impacts on work and occupational factors, and negative social consequences [10].

In decision-making regarding CT enrollment and participation, parents must contend with the complexity of medical information in stressful and potentially overwhelming circumstances [11, 12]. Additionally, CTs are often the only treatment option available to children with RDs. Therefore, every possible participant must be given the best chance of inclusion; all potential barriers to participation (including barriers related to knowledge needs and preferences) must be actively addressed. Empowering parents with relevant, tailored information is a key aspect of supporting informed decision-making about their child’s health, treatment, and disease management. To our knowledge and based on the results of a preliminary search, there has been no scoping review conducted on the knowledge needs and preferences of parents of children with RDs regarding PRDCTs.

## Review methods/design

### Purpose and objectives of the scoping review

The objectives of this review are to (1) identify and summarize the existing published and gray literature that investigates parental knowledge needs and preferences regarding PRDCTs; (2) summarize key findings from this literature; and (3) identify knowledge gaps in this area of study to inform future research and resource development. Developing an understanding of the evidence base and identifying gaps in this field is a fundamental step towards meeting the needs of parents and enhancing the ethical integrity, feasibility, and effectiveness of PRDCTs. A separate environmental scan is in development to identify any existing knowledge translation or educational tools that have been developed to address these knowledge needs; this scan will allow identification of tools that may not meet the inclusion criteria of this review (i.e., tools that have been developed with no association to a research study).

### Methods

We will conduct a scoping review based on Arksey and O’Malley’s [13] framework with enhancements by Levac et al. [14] and Peters et al. [15]. Scoping review methodology was selected due to the broad nature of the review question and scope of the review, and the intended purpose (i.e., determining the current state of knowledge on this topic and mapping the evidence) [13, 16]. The review will be reported in accordance with the Preferred Reporting Items for Systematic Reviews and Meta-Analyses Extension for Scoping Reviews (PRISMA-ScR) reporting guidelines [17] to enhance transparency in methodological choices and increase the relevance of the review for decision-making [18].

There is increasing recognition that communities meant to benefit from research must be active participants in the research process. We will engage RareKids-CAN Parent Partners throughout the scoping review who will provide insights based on their lived and living experience as knowledge users with unique expertise. Engagement with parents will be conducted according to the JBI guidance for knowledge user engagement in scoping reviews [19]. RareKids-CAN Parent Partners have been involved with the conceptualization of the review and will be invited to partner on data interpretation, presentation, and dissemination of results [19].

### Stage 1: identifying the review question

The main review questions are as follows: (1) What are the knowledge needs and preferences of parents regarding PRDCTs? and (2) What studies currently exist to address these needs and preferences? These questions were developed in collaboration with the RareKids-CAN Knowledge Mobilization (KM) Sub-platform, a group of parents, researchers, and healthcare providers within the RareKids-CAN network. The PCC (Population, Concept, Context) framework was used to guide question development [18].

#### Population

Studies that consider the knowledge needs and preferences of parents of children with a RD will be included in this review. Parents will be defined as the child’s primary caregiver (i.e., biological or adoptive parent, or legal guardian). There is no universal definition of a RD; for the purpose of this review, the European definition will be used (i.e., prevalence of <1 per 2000 people [1]) as it is the definition most used in Canada.

#### Concept

##### Knowledge needs and preferences

Knowledge needs have been defined as any gap in understanding a parent needs to address with data, facts, or ideas from an information source in order to make an informed decision. Knowledge preferences have been defined as parents’ preferred format, depth, context, and source of data, facts, and ideas. Parents’ knowledge needs and preferences regarding PRDCTs before, during, and after trial participation will be considered.

##### Clinical trials

CTs have been defined as any study evaluating the safety and/or effects of one or more treatments or interventions [20]. Treatments or interventions will be defined as any care provided to a patient to prevent, treat, or manage their RD, including but not limited to drugs, procedures, devices, surgical intervention, and types of therapy (i.e., physical, occupational).

#### Context

There will be no limits applied regarding the context of included literature in this review.

### Stage 2: identifying relevant studies

#### Search strategy and information sources

A systematic literature search (see Additional file 1) will be conducted with support from a research librarian to identify all relevant primary studies in the following databases: MEDLINE via Ovid, CINAHL Plus with Full Text via EBSCOhost, EMBASE via Ovid, PsycINFO via Ovid, Scopus via Elsevier, and Web of Science via Clarivate. Databases will be searched using keywords and controlled terms (subject headings), where available, for five main concepts: parents, knowledge needs/preferences, pediatrics, rare disease, and clinical trials. To reduce the likelihood of omitting a relevant article, the reference list of all included studies will be searched. Google and Perplexity AI, and the ProQuest Dissertations & Theses Global database via Clarivate will be used to search for gray literature (e.g., abstracts, conference proceedings, theses, dissertations, and reports on studies of relevance to this review) (see Additional file 2). The first 100 results of each search will be reviewed. We will also conduct a search within organizations’ websites that relate to pediatric RDs, such as the National Organization of Rare Disorders [21] and the National Institutes of Health Genetic and Rare Diseases Information Center [22].

Terms will be entered as text words and subheadings. Text words will be truncated, and terms will be combined using Boolean operators. Due to the large number of RDs and lack of indexed search terms related to RDs, both general search terms related to RDs and a list of specific pediatric RDs (collated by RareKids-CAN researchers [23]) will be included. The search was originally developed in MEDLINE via Ovid, then translated to be used within each additional database. Keywords will remain consistent while subject headings will vary marginally across databases due to differences in operational functions of each database.

### Stage 3: study selection

Search results will be exported to Covidence, a web-based citation manager, where duplicates will be removed. Study selection will then be divided into two phases: title/abstract screening, then full-text screening. We will pilot test the search strategy and select three to five studies retrieved from the original search; we will conduct abstract and full-text screening of these studies to identify any further refinements needed before conducting the finalized search strategy [14]. Articles will be independently screened by two reviewers according to detailed inclusion and exclusion criteria (Table 1) using an eligibility and screening criteria form (see Additional file 3). Reviewers will meet prior to, at the midpoint, and in the final stages of the abstract review process to discuss any questions and challenges; based on these discussions, search strategy refinements will be incorporated if required [14].

**Table 1:** Inclusion and exclusion criteria

Screening criteria	Inclusion criteria	Exclusion criteria
Study design/source of evidence	Primary research, any design (i.e., qualitative, quantitative, multi-method, and mixed methods studies) Secondary analysis of primary data Theses and dissertations	Reviews/knowledge syntheses Letters Opinion pieces Editorials Conference meeting abstracts/posters Published abstracts Commentaries Study protocols Websites Blogs Pamphlets Magazines Clinical guidelines Scientific reports/statements Association/organization reports/statements Books Book chapters Social media posts
Study focus	Parent knowledge needs and/or preferences re: PRDCTs	Parent knowledge needs and/or preferences were not included/explored in the inquiry
Study participants	Parents/guardians of a child with a RD*	Parents/guardians not included as participants in study
Study outcomes	Knowledge needs and/or preferences of parents regarding PRDCTs	No reference to knowledge needs and/or preferences of parents re: PRDCTs
Language in which study is reported	English	Not English
Setting	All countries	N/A
Year study was published	Any date	N/A

^*^RD: defined as a disease with a prevalence of <1 per 2000

All studies that meet the inclusion criteria will be considered regardless of study design or country of study/publication. Only studies in English will be included due to resource limitations of the review. Conference abstracts and posters will be excluded as they are likely to lack the required information to facilitate assessment of the design, methods, outcomes, and results of the study [24]. Reviews and knowledge syntheses will be excluded; however, we will scan the reference lists of any that are identified through the search to identify potentially relevant studies. During screening, articles will be classified as “include,” “exclude,” or “unclear” based on title and abstract review. Full texts for articles classified as “include” and “unclear” will be screened, and eligibility determined. Disagreements will be resolved by consensus and assessment by a third reviewer if required.

#### Inclusion and exclusion criteria

Inclusion and exclusion criteria are listed in Table 1, and explanations of certain criteria are expanded upon below.

##### Population

a Parent

Studies that include but are not limited to parents as participants (e.g., information needs of both health care providers and parents are examined) will be included during abstract screening, then assessed for eligibility at full-text screening; at this stage, if subgroup analysis data for the different populations is provided, the study will be included.

b Age of child

Studies investigating parents of children (21 years and younger) will be included. The broad age range of 0–21 years [25] was selected to facilitate inclusion of relevant literature involving older adolescents, and because 21 is the oldest age of majority globally. Studies involving a population of both children and adults (22 years and older) will be included if subgroup analysis has been conducted for each group; for instance, if both adult participants and parents of pediatric participants are included in a survey study about their experience within a RD CT, it will be included if subgroup data was analyzed and reported. While the focus of our review is parents’ knowledge needs and preferences in PRDCTs, it is common for RD research to include both pediatric and adult populations due to small sample sizes. Studies investigating only adult populations will be excluded.

iii. Child with a RD

Eligibility according to this criterion will be assessed by accessing the Orphanet RD database [26] or list of RDs published by the National Institutes of Health Genetic and Rare Diseases Information Center [22]. Studies with populations with multiple diagnoses will be excluded if no specific diagnoses are listed; however, if specific diagnoses are listed and any of them are a RD, the study will be included. Studies investigating a population with a diagnosis that has some rare and some non-rare forms will only be included if the rare form of the diagnosis is referenced, as it cannot be assumed that the participants had the rare form.

##### Concept

a CTs

Studies investigating parents’ knowledge needs and preferences regarding a wider range of clinical research (e.g., case-control studies, non-interventional research designs) will be included in the first stage of screening. Eligibility for inclusion will be confirmed at full-text screening; if knowledge needs and preferences regarding CTs are reported, the study will be included, and the details of the concept under investigation in these studies will be made explicit in the reporting of results to enhance interpretation of the findings.

##### Context

The context will not be limited by geographic location; studies from any country or region and across all relevant settings will be considered for inclusion.

#### Quality appraisal

The Mixed Methods Appraisal Tool (MMAT) version 2018 [27] will be used to assess the methodological quality of the studies included in this review. The MMAT is a checklist that includes criteria to appraise the quality of a study and has five sets of criteria for qualitative, randomized controlled trials, nonrandomized, quantitative descriptive, and mixed methods studies [27]. It acknowledges the distinct methodological characteristics of studies and means researchers can use one tool (as opposed to multiple tools according to study design) within a mixed methods review. It is therefore appropriate for this review, which will include heterogeneous study designs.

Quality assessment will be conducted by two reviewers independently. Reviewers will pilot the MMAT on three to five studies, then meet to compare results and confirm consistency of assessments. Disagreements in quality assessment will be resolved through discussion.

### Stage 4: charting the data

Data extraction will include details regarding participants, setting/context, concepts studied, study design, methods, and key findings related to the review questions. Two reviewers will use a data extraction form (see Additional file 4) to independently extract data, and then meet to compare findings. Study authors will be contacted if reviewers find unreported or unclear data during the extraction process. Disagreements between reviewers will be resolved through discussion. Extraction will be piloted on three to five studies to assess consistency between reviewers; refinements to the extraction form will be incorporated if needed [14].

### Stage 5: collating, summarizing, and reporting the results

This stage will involve mapping the main sources and types of evidence available. One reviewer will analyze the data; these preliminary findings will be shared with and reviewed by the research team and RareKids-CAN Parent Partners prior to summarizing the data. Their interpretations of the findings will be incorporated in the summary prior to finalizing the results. All studies regardless of methodological quality will be included in the synthesis.

#### Analyzing the data

Analysis will involve a descriptive numerical summary and qualitative content analysis. Characteristics of included studies (e.g., study design, year of publication) will be synthesized within an evidence table. Data related to key findings (i.e., parental knowledge needs and preferences related to PRDCTs) will be analyzed using conventional qualitative content analytical techniques [28]. We will map the identified themes to summarize parental knowledge needs and preferences emerging from the literature identified in the review.

#### Reporting results

Results will be reported as follows: an evidence table including the number and characteristics of included studies; themes identified from the analysis; and a narrative description of the quality of included studies and gaps in the evidence.

#### Applying meaning to results

Levac et al. [14] advocate for considering the implications of findings within the broader context to advance the legitimacy of scoping review methodology. We will consider the meaning of our results and the broader implications for policy, practice, and future research. The RareKids-CAN KM Sub-platform aims to support and accelerate KM within PRDCTs in Canada, which includes identifying the knowledge needs and preferences of knowledge users. The findings from this review will inform the co-creation of resources and tools based on these identified needs and preferences.

### Stage 6: consultation

Contributors to this consultation process will include RareKids-CAN Parent Partners and the RareKids-CAN KM Sub-platform. Their input will provide valuable insights regarding the findings of the review. There is a lack of clarity regarding how, when, and for what purpose to consult with interested parties in a scoping review; however, Levac et al. [14] recommend informing this consultation based on the findings from stage 5 to allow interested parties to build on the evidence and provide expertise, clarity in meaning, and perspective to the preliminary findings of the review. Consultations will include discussion with RareKids-CAN Parent Partners and the KM Sub-platform, in which we will share preliminary findings of the review and seek input (e.g., initial thoughts on the findings, any additional knowledge needs or preferences they can identify). Insights and feedback from these consultations will be integrated within the results and discussion in the final manuscript. This stage of the scoping review also constitutes part of the KM strategy; in the sharing of preliminary findings to interested parties, we are sharing findings with the RD community and will likely gain further understanding to inform the development of effective dissemination strategies of the overall findings of the review [14].

## Discussion

We will use rigorous, transparent methods to comprehensively identify, analyze, and summarize the relevant literature regarding parents’ knowledge needs and preferences regarding PRDCTs. We will map and present an overview of existing literature in this area. This review contributes to the emerging field of improving access to and experiences of families in PRDCTs and can guide future research, practices, and the development of knowledge translation tools to support parents of children with RDs. The findings from this review will inform a qualitative interview study with parents of children with a RD to explore their PRDCT knowledge needs and preferences. Findings will support the development of CT processes and sources of CT information to better support families affected by RDs and ultimately support the generation of new knowledge and treatments for pediatric RDs.

## Supplementary Information

Additional file 1: Search strategy for library databases.

Additional file 2: Search strategy for gray literature.

Additional file 3: Eligibility and screening criteria form.

Additional file 4: Data extraction form.

## Data Availability

Not applicable.

## Abbreviations

RD: Rare disease
CT: Clinical trial
PRDCT: Pediatric rare disease clinical trial

## Declarations

Ethics approval and consent to participate

Not applicable.
