# Supplementary material for: Identifying the knowledge needs and preferences of parents of children with rare diseases regarding clinical trials: a scoping review protocol
Source: Syst Rev. 2026 Feb 5;15:74. doi: 10.1186/s13643-026-03094-0 (PMC12964642; doi:10.1186/s13643-026-03094-0)
Supplement: Supplementary file 1 — Additional file 1: Search strategy for library databases. [file 13643_2026_3094_MOESM1_ESM.pdf]

Additional File 1

Search Strategy for Library Databases

MEDLINE

Ovid MEDLINE(R) ALL <1946 to August 11, 2025>

1 Parenting/ 23673

2 exp Parents/ 157443

3 Caregivers/ 58584

4 Parent-Child Relations/ 38247

5 (parent* or mother* or father* or caregiver* or guardian* or mom? or dad? or family or families or care giver* or caregiver* or carer*).mp. 2275833

6 1 or 2 or 3 or 4 or 5 2276453

7 exp Pediatrics/ 65600

8 (Child* or pediatric* or paediatric* or teen* or adolesc* or youth* or toddler* or infant* or neonate* or newborn*).mp. 5100368

9 7 or 8 5101514

10 exp Health Knowledge, Attitudes, Practice/ 138758

11 exp Consumer Health Information/ 16406

12 exp Information Seeking Behavior/ 3630

13 exp Information Sources/ 1415860

14 exp Consumer Health Informatics/ 154

15 exp Patient Education as Topic/ 91385

16 information needs.mp. 6408

17 knowledge needs.mp. 410

18 ((patient* or consumer* or stakeholder* or user* or lay* or client* or citizen* or communit* or public or advoca* or carer* or caregiver* or surrogate* or parent* or relative or mother* or father* or guardian*) adj3 (involv* or represent* or consult* or contribut* or engage* or activat* or opinion* or dialog* or partner* or input* or satisf* or experience* or communicat* or "decision-making" or "decision making" or knowledge)).mp. [mp=title, book title, abstract, original title, name of substance word, subject heading word, floating sub-heading word, keyword heading word, organism supplementary concept word, protocol supplementary concept word, rare

disease supplementary concept word, unique identifier, synonyms, population supplementary concept word, anatomy supplementary concept word] 880186

19 10 or 11 or 12 or 13 or 14 or 15 or 16 or 17 or 18 2445348

20 exp Rare Diseases/ 15319

21 (rare disease* or rare disorder* or rare diagnos* or rare condition* or orphan dis* or neglected disease or neglected disorder).mp. [mp=title, book title, abstract, original title, name of substance word, subject heading word, floating sub-heading word, keyword heading word, organism supplementary concept word, protocol supplementary concept word, rare disease supplementary concept word, unique identifier, synonyms, population supplementary concept word, anatomy supplementary concept word] 91771

22 ("AADC Deficiency" or "Acute lymphoblastic leukemia" or "Adenosine deaminase severe combined immunodeficiency" or "Aicardi Goutieres Syndrome" or "Alpha-Mannosidosis" or "Ataxia-Telangiectasia" or "ATP1A3 Disorders" or "Batten disease" or "Biotinidase Deficiency" or "Cystic Fibrosis" or "Cystinosis" or "Duchenne muscular dystrophy" or "Fabry disease" or "Familial hypercholesterolemia" or "Fragile X Syndrome" or "Friedreich ataxia" or "Gaucher Disease" or "Giant Axonal Neuropathy" or "Hemophagocytic lymphohistiocytosis" or "Hemophilia A" or "Hemophilia B" or "Hereditary angioedema" or "Hypophosphatasia" or "Infantile Krabbe Disease" or "Infantile neuroaxonal dystrophy" or "Juvenile idiopathic arthritis" or "Lymphoma" or "Lysosomal acid lipase deficiency" or "Molybdenum Cofactor Deficiency" or "Mucopolysaccharidosis" or "Mycosis fungoides" or "Sezary syndrome" or "Neonatal-onset multisystem inflammatory disease" or "Neuromyelitis optica spectrum disorder" or "NMOSD" or "Niemann-Pick Disease Type C" or "Pantothenate kinase-associated neurodegeneration" or "Phenylketonuria or Pompe disease or Primary hyperoxaluria type 1 or Primary insulin-like-growth-factor-1 deficiency or primary IGFD or Retinal dystrophy or Rett Syndrome" or "Severe combined Immunodeficiencies" or "Sickle cell disease" or "Spinal muscular atrophy" or "Tay-Sachs Disease" or "Tuberous Sclerosis" or "Tyrosinemia type 1" or "Van Hippel-Lindau" or "Wilson's disease" or "X-linked Adrenoleukodystrophy").mp. [mp=title, book title, abstract, original title, name of substance word, subject heading word, floating sub-heading word, keyword heading word, organism supplementary concept word, protocol supplementary concept word, rare disease supplementary concept word, unique identifier, synonyms, population supplementary concept word, anatomy supplementary concept word] 532426

23 ("clinical trial*" or "clinical research" or "RCT" or "randomized controlled trial" or "drug trial").mp. [mp=title, book title, abstract, original title, name of substance word, subject heading word, floating sub-heading word, keyword heading word, organism supplementary concept word, protocol supplementary concept word, rare disease supplementary concept word, unique identifier, synonyms, population supplementary concept word, anatomy supplementary concept word] 1707136

24 22 and 23 38060

25 20 or 21 or 24 129342

26 6 and 9 and 19 and 25 961

27 limit 26 to english language 922

EMBASE

Embase <1974 to 2025 August 11>

1 exp parent/ 311349

2 exp caregiver/ 149025

3 (parent* or mother* or father* or caregiver* or guardian* or mom? or dad? or family or families or care giver* or caregiver* or carer*).mp. [mp=title, abstract, heading word, drug trade name, original title, device manufacturer, drug manufacturer, device trade name, keyword heading word, floating subheading word, candidate term word] 2865762

4 1 or 2 or 3 2902093

5 exp pediatrics/ 135614

6 (Child* or pediatric* or paediatric* or teen* or adolesc* or youth* or toddler* or infant* or neonate* or newborn*).mp. [mp=title, abstract, heading word, drug trade name, original title, device manufacturer, drug manufacturer, device trade name, keyword heading word, floating subheading word, candidate term word] 5462078

7 5 or 6 5463904

8 exp attitude to health/ 142195

9 exp consumer health information/ 4505

10 exp health behavior/ 557070

11 exp information seeking/ 7679

12 exp information source/ 5381

13 exp consumer health informatics/ 189

14 exp patient education/ 136569

15 information needs.mp. 8368

16 knowledge needs.mp. 537

17 ((patient* or consumer* or stakeholder* or user* or lay* or client* or citizen* or communit* or public or advoca* or carer* or caregiver* or surrogate* or parent* or relative or mother* or father* or guardian*) adj3 (involv* or represent* or consult* or contribut* or engage* or activat* or opinion* or dialog* or partner* or input* or satisf* or experience* or communicat* or "decision-

making" or "decision making" or knowledge)).mp. [mp=title, abstract, heading word, drug trade name, original title, device manufacturer, drug manufacturer, device trade name, keyword heading word, floating subheading word, candidate term word] 1340489

18 8 or 9 or 10 or 11 or 12 or 13 or 14 or 15 or 16 or 17 1965824

19 exp rare disease/ 54404

20 (rare disease* or rare disorder* or rare diagnos* or rare condition* or orphan dis* or neglected disease or neglected disorder).mp. [mp=title, abstract, heading word, drug trade name, original title, device manufacturer, drug manufacturer, device trade name, keyword heading word, floating subheading word, candidate term word] 160843

21 ("AADC Deficiency" or "Acute lymphoblastic leukemia" or "Adenosine deaminase severe combined immunodeficiency" or "Aicardi Goutieres Syndrome" or "Alpha-Mannosidosis" or "Ataxia-Telangiectasia" or "ATP1A3 Disorders" or "Batten disease" or "Biotinidase Deficiency" or "Cystic Fibrosis" or "Cystinosis" or "Duchenne muscular dystrophy" or "Fabry disease" or "Familial hypercholesterolemia" or "Fragile X Syndrome" or "Friedreich ataxia" or "Gaucher Disease" or "Giant Axonal Neuropathy" or "Hemophagocytic lymphohistiocytosis" or "Hemophilia A" or "Hemophilia B" or "Hereditary angioedema" or "Hypophosphatasia" or "Infantile Krabbe Disease" or "Infantile neuroaxonal dystrophy" or "Juvenile idiopathic arthritis" or "Lymphoma" or "Lysosomal acid lipase deficiency" or "Molybdenum Cofactor Deficiency" or "Mucopolysaccharidosis" or "Mycosis fungoides" or "Sezary syndrome" or "Neonatal-onset multisystem inflammatory disease" or "Neuromyelitis optica spectrum disorder" or "NMOSD" or "Niemann-Pick Disease Type C" or "Pantothenate kinase-associated neurodegeneration" or "Phenylketonuria or Pompe disease or Primary hyperoxaluria type 1 or Primary insulin-like-growth-factor-1 deficiency or primary IGFD or Retinal dystrophy or Rett Syndrome" or "Severe combined Immunodeficiencies" or "Sickle cell disease" or "Spinal muscular atrophy" or "Tay-Sachs Disease" or "Tuberous Sclerosis" or "Tyrosinemia type 1" or "Van Hippel-Lindau" or "Wilson's disease" or "X-linked Adrenoleukodystrophy").mp. [mp=title, abstract, heading word, drug trade name, original title, device manufacturer, drug manufacturer, device trade name, keyword heading word, floating subheading word, candidate term word] 897565

22 ("clinical trial*" or "clinical research" or "RCT" or "randomized controlled trial" or "drug trial").mp. [mp=title, abstract, heading word, drug trade name, original title, device manufacturer, drug manufacturer, device trade name, keyword heading word, floating subheading word, candidate term word] 3399188

23 21 and 22 130148

24 19 or 20 or 23 288952

25 4 and 7 and 18 and 24 1992

26 limit 25 to english language 1942

PSYCINFO

APA PsycInfo <1806 to August 2025 Week 1>

1 exp Parenting/ 138649

2 exp Parents/ 151469

3 exp Caregivers/ 42816

4 exp Parent Child Relations/ 82240

5 (parent* or mother* or father* or caregiver* or guardian* or mom? or dad? or family or families or care giver* or caregiver* or carer*).mp. [mp=title, abstract, heading word, table of contents, key concepts, original title, tests & measures, mesh word] 847884

6 1 or 2 or 3 or 4 or 5 853544

7 exp Pediatrics/ 35600

8 (Child* or pediatric* or paediatric* or teen* or adolesc* or youth* or toddler* or infant* or neonate* or newborn*).mp. [mp=title, abstract, heading word, table of contents, key concepts, original title, tests & measures, mesh word] 1378822

9 7 or 8 1378822

10 exp Health Knowledge/ 10416

11 exp Health Behavior/ 50295

12 exp Health Attitudes/ 28379

13 exp Information Seeking/ 8306

14 exp Health Information/ 4854

15 consumer health informatics.mp. 74

16 exp Client Education/ 5071

17 information needs.mp. 2352

18 knowledge needs.mp. 226

19 ((patient* or consumer* or stakeholder* or user* or lay* or client* or citizen* or communit* or public or advoca* or carer* or caregiver* or surrogate* or parent* or relative or mother* or father* or guardian*) adj3 (involv* or represent* or consult* or contribut* or engage* or activat* or opinion* or dialog* or partner* or input* or satisf* or experience* or communicat* or "decision-making" or "decision making" or knowledge)).mp. [mp=title, abstract, heading word, table of contents, key concepts, original title, tests & measures, mesh word] 315625

20 10 or 11 or 12 or 13 or 14 or 15 or 16 or 17 or 18 or 19 404557

21 (rare disease* or rare disorder* or rare diagnos* or rare condition* or orphan dis* or neglected disease or neglected disorder).mp. [mp=title, abstract, heading word, table of contents, key concepts, original title, tests & measures, mesh word] 2620

22 ("AADC Deficiency" or "Acute lymphoblastic leukemia" or "Adenosine deaminase severe combined immunodeficiency" or "Aicardi Goutieres Syndrome" or "Alpha-Mannosidosis" or "Ataxia-Telangiectasia" or "ATP1A3 Disorders" or "Batten disease" or "Biotinidase Deficiency" or "Cystic Fibrosis" or "Cystinosis" or "Duchenne muscular dystrophy" or "Fabry disease" or "Familial hypercholesterolemia" or "Fragile X Syndrome" or "Friedreich ataxia" or "Gaucher Disease" or "Giant Axonal Neuropathy" or "Hemophagocytic lymphohistiocytosis" or "Hemophilia A" or "Hemophilia B" or "Hereditary angioedema" or "Hypophosphatasia" or "Infantile Krabbe Disease" or "Infantile neuroaxonal dystrophy" or "Juvenile idiopathic arthritis" or "Lymphoma" or "Lysosomal acid lipase deficiency" or "Molybdenum Cofactor Deficiency" or "Mucopolysaccharidosis" or "Mycosis fungoides" or "Sezary syndrome" or "Neonatal-onset multisystem inflammatory disease" or "Neuromyelitis optica spectrum disorder" or "NMOSD" or "Niemann-Pick Disease Type C" or "Pantothenate kinase-associated neurodegeneration" or "Phenylketonuria or Pompe disease or Primary hyperoxaluria type 1 or Primary insulin-like-growth-factor-1 deficiency or primary IGFD or Retinal dystrophy or Rett Syndrome" or "Severe combined Immunodeficiencies" or "Sickle cell disease" or "Spinal muscular atrophy" or "Tay-Sachs Disease" or "Tuberous Sclerosis" or "Tyrosinemia type 1" or "Van Hippel-Lindau" or "Wilson's disease" or "X-linked Adrenoleukodystrophy").mp. [mp=title, abstract, heading word, table of contents, key concepts, original title, tests & measures, mesh word] 13590

23 ("clinical trial*" or "clinical research" or "RCT" or "randomized controlled trial" or "drug trial").mp. [mp=title, abstract, heading word, table of contents, key concepts, original title, tests & measures, mesh word] 98259

24 22 and 23 655

25 21 or 24 3248

26 6 and 9 and 20 and 25 127

27 limit 26 to english language 114

CINAHL

Search History

S5 AND S8 AND S18 AND S24	**Limiters** - English Language<br><br>**Search modes** - Find all my search terms	371
S25	S5 AND S8 AND S18 AND S24	377

S24	S19 OR S20 OR S23	114,486
S23	S21 AND S22	5,857
S22	"clinical trial*" or "clinical research" or "RCT" or "randomized controlled trial" or "drug trial"	389,570
S21	"AADC Deficiency" or "Acute lymphoblastic leukemia" or "Adenosine deaminase severe combined immunodeficiency" or "Aicardi Goutieres Syndrome" or "Alpha-Mannosidosis" or "Ataxia-Telangiectasia" or "ATP1A3 Disorders" or "Batten disease" or "Biotinidase Deficiency" or "Cystic Fibrosis" or "Cystinosis" or "Duchenne muscular dystrophy" or "Fabry disease" or "Familial hypercholesterolemia" or "Fragile X Syndrome" or "Friedreich ataxia" or "Gaucher Disease" or "Giant Axonal Neuropathy" or "Hemophagocyt ...	79,265
S20	rare disease* or rare disorder* or rare diagnos* or rare condition* or orphan dis* or neglected disease or neglected disorder	108,949
S19	(MH "Rare Diseases")	6,292
S18	S9 OR S10 OR S11 OR S12 OR S13 OR S14 OR S15 OR S16 OR S17	1,217,618
S17	(patient* or consumer* or stakeholder* or user* or lay* or client* or citizen* or communit* or public or advoca* or carer* or caregiver* or surrogate* or parent* or relative or mother* or father* or guardian*) N3 (involv* or represent* or consult* or contribut* or engage* or activat* or opinion* or dialog* or partner* or input* or satisf* or experience* or communicat* or "decision-making" or "decision making" or knowledge)	418,631
S16	knowledge needs	71,126
S15	(MH "Patient Education+")	92,546
S14	(MH "Information Resources+")	622,203

S13	(MH "Information Seeking Behavior")	5,596
S12	(MH "Information Needs")	12,989
S11	(MH "Consumer Health Information+")	24,171
S10	(MH "Attitude to Health+")	195,700
S9	(MH "Health Knowledge")	45,341
S8	S6 OR S7	444,334
S7	child* or pediatric* or paediatric* or teen* or adolesc* or youth* or toddler* or infant* or neonate* or newborn*	1,568,538
S6	(MH "Pediatrics+")	23,805
S5	S1 OR S2 OR S3 OR S4	734,438
S4	parent* or mother* or father* or caregiver* or guardian* or mom# or dad# or family or families or care giver* or caregiver* or carer*	733,395
S3	(MH "Parent-Child Relations+")	44,796
S2	(MH "Caregivers")	49,415
S1	(MH "Parents+")	132,702

WEB OF SCIENCE

1,034 results from All Databases for:

parent* OR mother* OR father* OR caregiver* OR guardian* OR mom? OR dad? OR family OR families OR "care giver*" OR caregiver* OR carer*

AND

pediatric* OR paediatric* OR child* OR teen* OR adolesc* OR youth* OR toddler* OR infant* OR neonate* OR newborn*

AND

"rare disease*" OR "rare disorder*" OR "rare diagnos*" OR "rare condition*" OR "orphan dis*" OR "neglected disease" OR "neglected disorder"

AND

"health knowledge" OR "information sources" OR "patient education" OR "information seeking behaviour" OR "information seeking behavior" OR "information needs" OR "knowledge needs" OR ((patient* OR consumer* OR stakeholder* OR user* OR lay* OR client* OR citizen* OR communit* OR public OR advoca* OR carer* OR caregiver* OR surrogate* OR parent* OR relative OR mother* OR father* OR guardian*) NEAR/3 (involv* OR represent* OR consult* OR contribut* OR engage* OR activat* OR opinion* OR dialog* OR partner* OR input* OR satisf* OR experience* OR communicat* OR "decision-making" OR "decision making" OR knowledge))

Refined By: Languages: English

SCOPUS

833 documents found

parent* OR mother* OR father* OR caregiver* OR guardian* OR mom# OR dad# OR family OR families OR "care giver*" OR caregiver* OR carer*

AND

pediatric* OR paediatric* OR child* OR teen* OR adolesc* OR youth* OR toddler* OR infant* OR neonate* OR newborn*

AND

"rare disease*" OR "rare disorder*" OR "rare diagnos*" OR "rare condition*" OR "orphan dis*" OR "neglected disease" OR "neglected disorder"

AND

"health knowledge" OR "information sources" OR "patient education" OR "information seeking behaviour" OR "information seeking behavior" OR "information needs" OR "knowledge needs" OR ((patient* OR consumer* OR stakeholder* OR user* OR lay* OR client* OR citizen* OR communit* OR public OR advoca* OR carer* OR caregiver* OR surrogate* OR parent* OR relative OR mother* OR father* OR guardian*) W/3 (involv* OR represent* OR consult* OR contribut* OR engage* OR activat* OR opinion* OR dialog* OR partner* OR input* OR satisf* OR experience* OR communicat* OR "decision-making" OR "decision making" OR knowledge))

Limited to: English
