# Supplementary material for: Identifying the knowledge needs and preferences of parents of children with rare diseases regarding clinical trials: a scoping review protocol
Source: Syst Rev. 2026 Feb 5;15:74. doi: 10.1186/s13643-026-03094-0 (PMC12964642; doi:10.1186/s13643-026-03094-0)
Supplement: Supplementary file 2 — Additional file 2: Search strategy for gray literature. [file 13643_2026_3094_MOESM2_ESM.pdf]

Additional File 2

Search Strategy for Gray Literature

Search Strategy for Google Advanced Search

1. (parent* OR caregiver*) AND (“rare disease*” OR “orphan disease*” OR “rare disorder*” OR “rare diagnosis*” OR “rare condition*”) AND (“information need*” OR “knowledge need*” OR “information preference*” OR “knowledge preference*”)
2. (parent* OR caregiver*) AND (“rare disease*” OR “orphan disease*” OR “rare disorder*” OR “rare diagnosis*” OR “rare condition*”) AND “clinical trial” AND (experience* OR satisfaction* OR decision-making OR “decision making” OR knowledge OR information OR opinion*)

Search Strategy for Perplexity AI

1. “What are the knowledge needs and preferences of parents regarding pediatric rare disease clinical trials?”
2. “What are parents’ informational needs about clinical trials for rare diseases?”
3. “What do parents want and need to know about pediatric rare disease clinical trials?”

ProQuest Dissertations & Theses Global database via Clarivate

1. (parent* OR caregiver*) AND (“rare disease*” OR “orphan disease*” OR “rare disorder*” OR “rare diagnosis*” OR “rare condition*”) AND (“information need*” OR “knowledge need*” OR “information preference*” OR “knowledge preference*”)
2. (parent* OR caregiver*) AND (“rare disease*” OR “orphan disease*” OR “rare disorder*” OR “rare diagnosis*” OR “rare condition*”) AND “clinical trial” AND (experience* OR satisfaction* OR decision-making OR “decision making” OR knowledge OR information OR opinion*)
