# Supplementary material for: Identifying the knowledge needs and preferences of parents of children with rare diseases regarding clinical trials: a scoping review protocol
Source: Syst Rev. 2026 Feb 5;15:74. doi: 10.1186/s13643-026-03094-0 (PMC12964642; doi:10.1186/s13643-026-03094-0)
Supplement: Supplementary file 3 — Additional file 3: Eligibility and screening criteria form. [file 13643_2026_3094_MOESM3_ESM.pdf]

Additional File 3

Eligibility and Screening Criteria Form

Scoping Review Eligibility and Screening Criteria

RESEARCH QUESTION & SEARCH PARAMETER

RESEARCH QUESTION:
What are the knowledge needs and preferences of parents regarding pediatric rare disease clinical trials (PRDCTs)?

RESTRICTIONS:
English language, no restrictions on date of publication, no restrictions on location of conduct/publication of study

LIMITS RE: STUDY DESIGN
Include: all primary research studies (any study design)
Exclude: reviews/knowledge syntheses*, letters, opinion pieces, editorials, conference meeting abstracts, posters, published abstracts, commentaries, study protocols, websites, blogs, pamphlets, magazines, clinical guidelines, scientific reports/statements, association/ organization, reports/statements, books, book chapters, social media posts
**Flag potentially relevant reviews/knowledge syntheses for hand-searching of reference lists*

DATA SOURCES

- Databases: MEDLINE via Ovid, CINAHL via EBSCOhost, Embase via Ovid, PyschINFO via Ovid, Scopus via Elsevier, Web of Science via Clarivate
- Grey literature: Google, Perplexity AI, ProQuest Dissertations & Theses Global database via Clarivate
- Websites of relevant rare disease organizations (e.g., Canadian Organization of Rare Diseases and the National Institutes of Health Genetic and Rare Diseases Information Center)
- Other: pursuing references in included articles

SEARCH FILTERS (if applicable):
English language

SCREENING QUESTIONS	INCLUSION CRITERIA	EXCLUSION CRITERIA
**Is the study reported in English?**<br>***ABSTRACT SCREENING***<br><br>**Yes/No/Unclear**	- Reported in English	- Not reported in English
**Does this study involve parents of a child with a rare disease (RD)?**<br>***ABSTRACT SCREENING***<br><br>**Yes/No/Unclear**	**Population:**<br>- Parents of children/youth (0-21 years) with a RD**<br>- Study population has multiple diagnoses which are reported in the study, and at least one is a RD	- Parents of adult children (i.e., the parent's child was >22 years at the time of the study)<br>- Study population has multiple diagnoses, but specific diagnoses not reported
**Does this study investigate parent experience with their child's RD (treatment, management, care, research, etc.)?**<br>***ABSTRACT SCREENING***<br><br>**Yes/No/Unclear**	**Concept:**<br>- Parent experiences, views, perceptions, attitudes, beliefs re: their child's RD	- Investigation is not concerned with parent perspective<br>- No investigation of parent experiences, views, perceptions, attitudes, beliefs re: their child's RD

**Is the type of evidence source one of the following:**<br>***ABSTRACT SCREENING***<br><br>**Yes/No/Unclear**	- Primary research, any design (i.e., qualitative, quantitative, multi-method, and mixed methods studies)<br>-Secondary analysis of primary data<br>- Theses and dissertations	- Reviews/knowledge syntheses<br>- Letters<br>- Opinion pieces<br>- Editorials<br>- Conference meeting abstracts/posters<br>- Published abstracts<br>- Commentaries<br>- Study protocols<br>- Websites<br>- Blogs<br>- Pamphlets<br>- Magazines<br>- Clinical guidelines<br>- Scientific reports/statements<br>- Association/organization reports/statements<br>- Books<br>- Book chapters<br>- Social media posts
**Does the study include investigation of/findings about parent knowledge needs and preferences re: PRDCTs?**<br>***FULL TEXT REVIEW***<br><br>**Yes/No/Unclear**	**Phenomenon of Interest:**<br>- Parent knowledge needs re: PRDCTs	- No reference to/inclusion of findings about parent knowledge needs re: PRDCTs
**FINAL DECISION:**<br><br>**Include/Exclude/Unclear**		
***Rare Disease: disease with prevalence of <1 per 2000 people*		
