# Supplementary material for: Identifying the knowledge needs and preferences of parents of children with rare diseases regarding clinical trials: a scoping review protocol
Source: Syst Rev. 2026 Feb 5;15:74. doi: 10.1186/s13643-026-03094-0 (PMC12964642; doi:10.1186/s13643-026-03094-0)
Supplement: Supplementary file 4 — Additional file 4: Data extraction form. [file 13643_2026_3094_MOESM4_ESM.pdf]

Additional File 4

Data Extraction Form

<table>
<tr><td colspan="3">Scoping Review Data Extraction Form</td></tr>
<tr><td colspan="3">REVIEW QUESTION</td></tr>
<tr><td colspan="3">What are the knowledge needs and preferences of parents regarding PRDCTs?</td></tr>
<tr><td colspan="3">REVIEW OBJECTIVES</td></tr>
<tr><td colspan="3">1. Identify and summarize the existing published and grey literature that investigates parental knowledge needs and preferences regarding PRDCTs;
2. Summarize key findings from this literature related to parental knowledge needs and preferences regarding PRDCTs;
3. Identify knowledge gaps in this area of study.</td></tr>
<tr><td>Section</td><td colspan="2">Data Extracted</td></tr>
<tr><td>1</td><td>REVIEWER</td><td>DATE OF EXTRACTION</td></tr>
<tr><td rowspan="3">2</td><td colspan="2">TITLE</td></tr>
<tr><td>AUTHOR(S) OF PUBLICATION</td><td>YEAR OF PUBLICATION</td></tr>
<tr><td>JOURNAL/SOURCE</td><td>COUNTRY</td></tr>
<tr><td>3</td><td colspan="2">TYPE OF STUDY
☐ Quantitative
☐ Qualitative
☐ Mixed Methods</td></tr>
<tr><td>4</td><td colspan="2">Study aim/objectives (i.e., purpose of study):</td></tr>
<tr><td rowspan="5">5</td><td colspan="2">Phenomena of interest:</td></tr>
<tr><td colspan="2">Study design/methodology/methods:</td></tr>
<tr><td colspan="2">Number of participants:</td></tr>
<tr><td colspan="2">Characteristics of participants:</td></tr>
<tr><td colspan="2">Setting and context-related information:</td></tr>
<tr><td>6</td><td colspan="2">Outcomes/Findings of significance to review objectives:</td></tr>
<tr><td>7</td><td colspan="2">Author interpretations/conclusions:</td></tr>
<tr><td>8</td><td colspan="2">Reviewer’s comments:</td></tr>
</table>

Adapted from the JBI methodology for scoping reviews (Peters MDJ, Godfrey C, McInerney P, Munn Z, Tricco AC, Khalil, H. Scoping Reviews (2020). Aromataris E, Lockwood C, Porritt K, Pilla B, Jordan Z, editors. JBI Manual for Evidence Synthesis. JBI; 2024. Available from: https://synthesismanual.jbi.global. https://doi.org/10.46658/JBIMES-24-09
